# Roles and Responsibilities in the Provision of Accredited Continuing Medical Education/Continuing Professional Development

**DOI:** 10.1080/21614083.2017.1314416

**Published:** 2017-05-04

**Authors:** Reinhard Griebenow, Craig Campbell, Graham T. McMahon, Kate Regnier, Jennifer Gordon, Eugene Pozniak, Daiana Stolz, Amir Qaseem, Gerd Antes, Samar Aboulsoud, Helmut König, Dirk Schulenburg, Hans Gehle, Peter Mills, Lampros Michalis, Heinz Weber, Robert Schaefer

**Affiliations:** ^a^ European Board for Accreditation in Cardiology (EBAC), Cologne, Germany; ^b^ European Cardiology Section Foundation (ECSF), Cologne, Germany; ^c^ Royal College of Physicians and Surgeons of Canada, Ottawa, Canada; ^d^ Accreditation Council for CME (ACCME), Chicago, USA; ^e^ Siyemi Learning, Manchester, UK; ^f^ Clinic for Pulmonary Medicine and Respiratory Cell Research, University Hospital Basel, Basel, Switzerland; ^g^ American College of Physicians (ACP), Philadelphia, PA, USA; ^h^ German Cochrane Center, Center for Medical Biometry and Medical Informatics, University of Freiburg, Freiburg, Germany; ^i^ Qatar Council for Healthcare Practitioners, Ministry of Public Health, Doha, Qatar; ^j^ Beiten Burkhardt Lawyers, Dusseldorf, Germany; ^k^ Chamber of Physicians North-Rhine, Duesseldorf, Germany; ^l^ Marburger Bund, Berlin, Germany

**Keywords:** Providers in CME, accredited CME, components constituent to accredited CME, procedural description of roles and responsibilities

## Abstract

The Cologne Consensus Conference 2015 has focused on “Providers in accredited CME[continuing medical education]/CPD [continuing professional development]”. As an outcome of the CCC 2015, the authors of this paper, who were part of the faculty, propose a contemporary definition of the roles and responsibilities of stakeholders involved in the different stages of planning, delivery and evaluation of CME/CPD.

## Introduction

As the needs and diversity of practice of physicians have evolved, so too has the need to evolve the planning, organisation and conduct of individual and group learning activities in the field of accredited continuing medical education (CME) and continuing professional development (CPD). This evolution requires the educational planner to assess the professional practice-based needs of physicians; be attentive to the evidence that informs practice [1]; incorporate interdisciplinary, [2] as well as inter-professional [3], education as needed; select an optimal method of learning; design outcome assessments; and account for the professional and/or legal regulations on planning and delivery of CME/CPD [4] to assure the activity is balanced and commercially independent. As CME/CPD continues its evolution with a goal of developing high-quality education delivered by medical experts to medical experts [5], CME/CPD is increasingly becoming a multi-professional enterprise [6], in which roles and responsibilities of physicians need to evolve in order to achieve this vision.

The aim of this paper is to propose a contemporary description of roles and responsibilities of stakeholders involved in the different stages of planning, delivery and evaluation of CME/CPD and to stimulate and guide discussions on defining roles and responsibilities for different stakeholder types within the evolving process of accredited CME/CPD.

The authors of this paper were part of the faculty at the Cologne Consensus Conference 2015, which focused on “Providers in accredited CME/CPD” [7].

## Process

International CME/CPD accreditation systems differ in their definition of a legitimate “provider”:

1) The Accreditation Council for Continuing Medical Education (ACCME) defines eligibility for accreditation as any entity that has a regular, recurring programme of CME, can meet the accreditation requirements, and is **not** a commercial interest, defined as “any entity producing, marketing, re-selling, or distributing health care goods or services consumed by, or used on, patients” [8]. The following types of organisations are accredited by the ACCME:

- Schools of medicine

- Medical specialty societies

- Insurance companies

- Medical education and publishing companies

- Government agencies

- Hospitals and health systems

- Foundations and other non-profit organisations

Providers are held “accountable for compliance with the accreditation requirements and the independence of CME” [8].

2) Other accreditation systems mandate that a provider of CME has to be “aligned with a …recognised specialty” and/or must be a “physician organisation”, defined as a “not-for-profit group of health professionals with a formal governance structure, accountable to and serving, among others, its specialist physician members through:

- continuing professional development,

- provision of health care, and/or

- research” [9].

3) In Europe, provision of CME is not legally considered a “service of general interest”, and is thereby exempt from competition law – i.e. accreditors have to take care that their rules do not distort competition [4]. Thus, medical education companies may provide accredited CME, and organisations may only be ineligible as a provider due to conflict of interest – e.g. pharmaceutical companies, medical device industry, etc. Individual nations may further modify the eligibility of provider types to participate in accredited CME using legislation and other methods [10].

The primary value of accreditation is to assure physicians that they are not being subject to promotion and marketing messaging, and that the education is developed to address an identified need of the profession, and is based on valid content; relevant to practice; evaluated for changes in competence, performance or patient outcomes; and independent of commercial interests. All accreditation frameworks adapt to applicable regional or national law, but must primarily ensure that all physicians involved in accredited CME are fully independent of any third party, and in particular of any commercial influence.

The type of organisation that is eligible to become a “provider” is of lesser importance to the overall context than is the expectation that the CME/CPD is uninfluenced by any undue commercial interests [8].

To attain this independence, individuals who participate in all parts of the planning of CME/CPD must be determined to be, themselves, independent of any third-party influence or commercial interest. Various mechanisms have evolved for managing and resolving disclosed commercial relationships, though some associations (e.g. employment by a commercial interest such as a pharmaceutical company) are considered irresolvable and preclude participation in the planning and presentation of the education.

To be inclusive and protective at the same time, there is a need to analyse and weight these roles, relationships and obligations of physicians involved in accredited CME/CPD. Different stakeholders involved in CME/CPD need to have defined mechanisms regulating their participation in the planning and delivery of CME/CPD. (Table 1).

**Table 1.** Components relevant for design, planning, conduct and evaluation of accredited CME/CPD.

**Why**
Needs assessment:
definition of source of information definition of assessment method conduct of assessment evaluation of assessment conclusion implementation of conclusions
**What (for)**
definition of content/general learning objectives/general
**Who and how**
educational format matching of educational format and content (agenda) nomination of faculty members declaration of conflict of interest of faculty members management of conflict of interest (final) selection of faculty content preparation (of individual presenter) learning objectives (per individual presenter) content presentation involvement in live interaction with participants (discussion, etc.) tests/choice of method (e.g. multiple choice questions, assessment of practical skills) test/set up test/conduct test/evaluation
**Feedback**
educational effectivity independence of information potential for change items to be evaluated evaluation format question design evaluation/conduct evaluation/analysis evaluation/conclusion evaluation/implementation of conclusions evaluation/publicising
**Logistics**
honoraria/reimbursement (travel, accommodation)/definition honoraria/reimbursement/transfer travel and accommodation announcement/marketing/press pre-/post-event registration room/technical equipment/food and drinks verification of attendance certificates of attendance/distribution sustainable materials (e.g. printed program)
**Sponsoring**
nomination of potential sponsors communication with sponsors (final) selection of sponsors exhibition/promotion/definition of type and place financial transfers
Accreditation procedure*
collection of all documents needed for application submission of application communication with accreditor (during accreditation process) appeals process certificates of attendance/layout

* applies only in case of event accreditation

In this regard, we are not proposing to introduce a new “universal definition” of a CPD provider, but have dissected which roles and responsibilities can be assumed by different stakeholder organisations involved in CME/CPD. In this process, we have used the term “overall responsibility” in the sense that only the physician(s) in charge – and nobody else – will be held responsible to the accreditor and also to participants, although the physician(s) may not have executed every single step of the respective process themselves (e.g. booking of hotel rooms, transfer of honoraria, etc.). Defining a stakeholder group as having “overall responsibility” is consistent with recommendations such as those developed by the Canadian Medical Association that “the ultimate decision on funding arrangements for CME/CPD activities is the responsibility of the physician-organizers.”[11] In contrast, “full responsibility” in this context means that in addition to what has been said under “overall responsibility”, the respective process has exclusively been executed by the physician(s) in charge (e.g. selection of content, speakers, etc.). In certain accreditation systems, such as the ACCME, there are no prescribed roles/responsibilities that can only be filled by physicians. Accredited providers are authorised to name the individuals who will act as their agents in ensuring that the requirements of accreditation are met. Therefore, in the following paragraphs, when referencing “physicians” and/or “physician organisations,” we are referring to agents of the accredited organisations.

## Proposal

We defined four major groups involved in the delivery of CME/CPD. This includes, (a) individual physicians, (b) physician organisations, (c) event organisers and (d) non-medical experts. Based on the data shown in Table 1, we propose the following procedural description of roles and responsibilities of these stakeholders for the major components of CME/CPD:

1. Needs assessment: This is the first step that defines the scope and objectives of a CME/CPD activity. According to current recommendations, a broad variety of sources maybe used to define a need. Since the methodological robustness of needs assessment is of crucial importance for the success of a CME/CPD activity, it is the responsibility of physicians and/or physician organisations to define acceptable sources of information, and draw and implement conclusions to initiate the process of planning CME/CPD activities. Data from external sources, such as professional organisers, may be used.
2. Content development and objectives: Defining the content and expected impact or outcome(s) of a CME/CPD activity may require advice from other professionals in relation to medico-legal affairs, biomedical ethics, communications or advocacy, but should otherwise fall under full responsibility of physicians and/or physician organisations.
3. Educational format: Selection of the optimal educational format for presentation of content and achievement of desired objectives may benefit from advice from both knowledgeable clinicians and non-medical experts. The overall responsibility remains with physicians and/or physician organisations, who must match the content to the educational format.
4. Choice of faculty: This starts with nomination of potential members and must include a process of conflict-of-interests management before final selection of faculty members. Patients; public representatives; professionals from nursing, pharmacy and other health disciplines; students; and clinicians of all types may be suitable faculty as planners and/or speakers in an effective educational programme. This again is the full responsibility of physicians and/or physician organisations, and includes critical steps such as defining the criteria for management of conflict of interest (including change of assignment of speakers to topics, or even exclusion) and subsequently making a final selection of faculty members.
5. Presentation of content: Preparation of the content of individual presentations, as well as the presentation itself, is managed by the speakers (physician, public representative, student, non-physician professional) themselves. The content of each speaker’s presentation may be subject to review by the programme leadership or appropriate designee (often a content specialist), as needed. However, it ultimately remains the full responsibility of the **individual** physician (or non-medical presenter), and this also applies to all forms of interactive involvement with the audience/participants. With regard to CME/CPD activities organised by a medical professional organisation, we think that some overall responsibility remains with the organisation, due to the fact that it has selected the faculty. All other stakeholders should have no role in this process.
6. Evaluating outcomes: Since assessment of the outcomes of CME/CPD activities is a common CPD accreditation standard to identify the “success” of the educational activity, the overall responsibility for evaluating outcomes remains with physicians and/or physician organisations, although the evaluation process may be supported or managed by others.
7. CPD Activity Review: Post-processing of CME/CPD activities is an integral part of quality assurance in accredited CME/CPD, not only in terms of addressing procedural variables, such as independence of information, transparency, effectiveness or capability of speakers, etc., but also as an indispensable source of information for needs assessment, as well as definition of future learning objectives, and feedback to organisers and accreditors. Thus, most of the components (evaluation format, question design, analysis of evaluation, etc.) of this process may have been advised by non-medical experts regarding set-up and design, though overall responsibility will remain with the physicians and/or physician organisations, which should take full responsibility for interpretation of results, conclusions drawn from the results and implementation of changes.
8. Selection of sponsors: Sponsorship decisions should be based on the principles of neutrality (equal chances for all sponsors), transparency, documentation (all items to be fixed in written contracts) and separation from engagement in roles that must be assumed by physicians. While transparency and documentation have increasingly been implemented, neutrality and separation of roles are still not sufficiently practised in all parts of the world. Regarding the distribution of roles, physicians should take overall responsibility for assuring neutrality, which includes opportunities for presentation of sponsors (industry exhibition). Transparency has to follow the principles of conflict-of-interests management or the requirements for sponsors as issued by accreditors. With regard to separation of roles, we consider it useful that all physicians playing an active part in the delivery of accredited CME/CPD should not be directly involved in communication with sponsors and/or execution of financial transfers. The ACCME, for example, requires separation of sponsorship from the selection of speakers or faculty, and dictates that sponsors may have no role in the selection of speakers, determination of content, editing or provision of materials, or influence of clinicians by reimbursing for their travel and attendance. Nevertheless, from our point of view, physicians and physician organisations will be associated with the choice of sponsors by both the profession itself and the public, and should thus take full responsibility for final selection of sponsors.
9. Logistics: In recent years, there has been an increasing awareness that the effect of a CME/CPD activity is dependent not only on its core content, but also on the context within which the educational process is supported in terms of its amenities, site layout, type of venue for the event/accommodation, design of communication materials, etc. These should thus fall under the overall responsibility of the physicians and/or physician organisations, with technical support from event organisers and non-medical experts. Learners should not receive stipends or travel awards from commercial sponsors, since this creates undue influence.
10. Accreditation procedure: Currently, most accreditation systems grant accreditation prior to the individual event. Thus, the accreditation procedure must provide sufficient proof or evidence that the requirements of the accreditor have been met. Though items to be demonstrated may vary from accreditor to accreditor, or depending on whether provider accreditation or event accreditation is practised, overall responsibility must always lie with the physicians and/or physician organisations.

## Discussion

According to the pedagogical principle that “effective CPD is contextual” and that “it is from immersion in practice that effective CPD arises” [5] all **medical** CME/CPD must in the end be expert education, delivered by medical experts to medical experts. This does not imply that non-medical experts (e.g. legal experts, educationalists, etc.) cannot contribute to this process on various levels during the planning, organisation, conduct or evaluation of CME/CPD in order to achieve optimal results. However, given the intended impact of education on patient care, the medical profession must ensure the credibility and expertise (i.e. professionalism) of statements and/or recommendations made during the CME/CPD activity (see e.g. [12]). Thus, whatever the division of roles and responsibilities in the planning and delivery of CME/CPD, this educational principle (“CPD for the profession, by the profession”) should supersede all other role models, and result in physicians assuming extensive responsibility for their ongoing CPD as part of professional self-regulation.

There are many situations in which shared responsibility with external groups or individuals is both inevitable and appropriate and does not compromise overall responsibility of the physician organiser. For example, an educationalist will be fully responsible for the professional quality and independence of their advice, but the overarching definition of the medical objectives to be achieved via implementation of the methods advised by the educationalist, as well as putting the results of this process into perspective, must be the sole responsibility of the physician organiser.

Consideration of roles and responsibilities also relates to all aspects of information dissemination and should not be restricted to the selection and presentation of content and choice of speakers. Thus, mainly due to recent transparency initiatives by industry, accreditors have provided detailed rules about where logos might be placed on information material; which wording has to be used to describe sponsoring, in contrast to corporate communication of the pharma- or medical device industry; etc. [13–16]. All this intends to provide participants with unambiguous information and prevent any misunderstandings about the process of selection of and participation in an activity, as well as post-processing of the activity.

With regard to the level of scrutiny to be applied, it may be helpful to refer to general principles of consumer protection as determined by applicable law: The European Court of Justice has recently made a decision on the “raspberry- vanilla- adventure”-tea case, ruling that applicable European law “must be interpreted as precluding the labelling of a foodstuff and methods used for the labelling from giving the impression, by means of the appearance, description or pictorial representation of a particular ingredient, that that ingredient is present, even though it is not in fact present and this is apparent solely from the list of ingredients on the foodstuff’s packaging” [17]. In this case, Teekanne, a tea manufacturer, marketed a fruit tea where the packaging suggested that it contained raspberry and vanilla, when no such constituents were present.

This applies to accredited CME/CPD, where an activity that (by accreditation) has to be free of any commercial influences on selection of content and speakers, as well as presentation of content, must not exhibit any elements that might cast doubt on this. This may be particularly relevant in countries with anti-corruption legislation, including specific regulations for medical professionals [18].

With regard to content, assigning full responsibility (in particular regarding scientific validity of content) to the medical profession is of particular importance in cases where content of CME/CPD is based on weak evidence (e.g. in “expert opinions” in clinical guidelines, or in relation to “alternative medicine” [19]).

It is beyond the scope of this paper to review all terminology in use around CME/CPD activities. However, we would like to stress that all roles and responsibilities should be clearly defined and assigned, and that definitions should ideally be publicly available. This relates in particular to terms that have a historical background without having been adapted to current requirements, e.g. the term “under the auspices of” (in most cases a scientific society), which may cover options ranging from reputational advantage to financial involvement or distinct roles in the process of developing and delivering a CME/CPD activity. Thus, unless terms and conditions for provision of such labelling have been clearly defined with regard to roles and responsibilities in the provision of accredited CME/CPD, we recommend abstaining from using such terms in order to avoid confusion.

With regard to provider vs. event accreditation, both systems aim to meet the same objectives (ACCME/RCPSCa/Germany/EBAC). Thus, regarding roles and responsibilities, as described in this article, contact partners, documentation procedures and control options may vary between both approaches to accreditation, but do not impact on the concept of roles and responsibilities as outlined above.

## Conclusion

Since it is difficult to provide a unifying definition of who might be a provider of accredited CME/CPD by type of organisation, we have instead proposed a set of components that are relevant to the design, planning, conduct and post-processing of accredited CME/CPD, for which the roles and responsibilities of stakeholders in accredited CME/CPD have been defined to satisfy accreditation standards. A principal model for medical CME/CPD is its delivery by knowledgeable independent professionals, which also contributes importantly to professional self-regulation. In addition, abstention from a definition of providers by type of organisation affects considerations regarding conflict of interest (of individuals or organisations), which now plays an important role in the decision on the eligibility of individuals, as well as organisations as providers, in accredited CME/CPD.

This approach should contribute to unambiguous expression in all communication between organisers and (potential) participants, in particular with regard to the independence of accredited CME/CPD from any undue third-party influence.

The concepts outlined in this article may gain even more importance if applicable legislation (e.g. anti-corruption law) becomes relevant to aspects of the accreditation process. The proposal above must now be discussed in the medical community and will probably need to be amended, in particular depending on the evolution of applicable law.

## Supplementary Material

Decalaration_of_interest.pdf
