# Supplementary material for: Roles and Responsibilities in the Provision of Accredited Continuing Medical Education/Continuing Professional Development
Source: J Eur CME. 2017 May 4;6(1):1314416. doi: 10.1080/21614083.2017.1314416 (PMC5843046; doi:10.1080/21614083.2017.1314416)
Supplement: Decalaration_of_interest.pdf [file ZJEC_A_1314416_SM8802.pdf]

Declaration of interests:

1. **Prof. Reinhard Griebenow, MD, PhD:**

   Financial: 0

   Nonfinancial: Assistant Medical Director and Head of Training, Department of Cardiology, Angiology and Diabetology, Municipal Hospital Cologne (Merheim), University of Cologne, Germany

   President, UEMS Cardiology Section

   Chairman of the Board, ECSF

   Chairman, EBAC Advisory Committee

   Head, Academy for Training and Education, Chamber of Physicians North-Rhine

   Member of the Board, Chamber of Physicians North-Rhine

   Member, Standing Committee for CME, German Medical Association

   Member, Education Committee, German Cardiac Society

   Member, Editorial Board, JECME

2. **Craig Campbell, MD**

   Financial: 0

   Nonfinancial: Director, Continuing Professional Development, Royal College of Physicians and Surgeons of Canada , Ottawa, Canada

Member, Medbiquitous board

4. **Amir Qaseem, MD, PhD, MHA, FACP**

   Financial: 0

   Nonfinancial:

Director, Clinical Policy, American College of Physicians, Philadelphia , USA

Member and Immediate Past President, Board of Trustees, Guidelines International Network

Member, Board of Directors, American Medical Association

PCPIF Co-chair, National Quality Forum, Health and Well Being Committee

Member, Measures Application Partnership

Member, Medbiquitous board

Member GRADE Working Group

Member DECIDE Advisory Board

Member, Board of Executives, DynaMed

5. **Jennifer Gordon, Med, CAE**

   Financial: 0

   Nonfinancial: Associate Director, Continuing Professional Development, Royal College of Physicians and Surgeons of Canada Member,

   Editorial Board, JECME

Member, Global Education Advisory Board, Merck Serono / Pfizer

6. **Prof. Lampros Michalis, MD, PhD**

   Financial: 2

   Nonfinancial:

   - **Member of the British Medical Association, UK**
   - **Member of the Royal College of Physicians, UK**
   - **Member of the British Society of Echocardiography**
   - **Regional Representative of the British Society of Echocardiography for the West Midlands region**
   - **Member of the Greek Cardiac Society**
   - **Member of the British Cardiac Society**
   - **Member of the Working Group of the Greek Cardiac Society for Interventional Cardiology**
   - **Fellow of the European Society of Cardiology (FESC)**
   - **Member of the Scientific Councils of Atherosclerosis/ Thrombosis and Vascular Biology of the American Heart Association (AHA)**
   - **Member of the International Society of Endovascular Specialists**
   - **National Representative of the Hellenic Cardiology Society to the UEMS Cardiology Section.**

- Professional Member of the American Heart Association and American Stroke Association.

- Member of the Greek Society of Cardiovascular Research

- Member of the Working Group of Aortic Disease and Peripherals of the Hellenic Cardiology Society

- Director of the Executive Committee of the Michaelideion Cardiac Center

- Member of the Board of Directors of the Hellenic Cardiological Society

- Appointed Member of the Hellenic Cardiological Society

- Nucleus Member of the Working Group of Invasive Cardiology of the Hellenic Cardiology Society

- President of the working group of Invasive Cardiology of the Hellenic Cardiology Society

- Vice president of the Scientific Committee of the Greek College of Cardiology

- Member of the European Board for Accreditation in Cardiology Reviewing Committee

- Member of the Executive Board of the University Hospital of Ioannina

- Secretary of the UEMS cardiology section – Vice President of EBAC

- Member of the Board of the ECSF (European Cardiology Section Foundation)

- Chief Editor of the Continuing Education of Cardiology Journal

of the Greek College of Cardiology

- Member of the Public Relations Committee of the European Society of Cardiology

- Substitute member of the Committee responsible for the examinations certifying the Completion of Specialist Training in Cardiology in the region of Epirus

- In charge of the Scientific Committee of the Research Laboratory of the University Hospital of Ioannina

- Member of the Committee responsible for the examinations certifying the Completion of Specialist Training in Cardiology in the region of Epirus

- Substitute member of the Educational Committee of the Greek National Board of Health

- Member of the Greek NHS Committee for the Evaluation of Cardiac Centers capability in regards with the implantation of pacemakers and defibrillators.

- Member of EBAC Advisory Committee

- Member of the International Scientific Committee organizing actions for the attraction of Scientific Meetings in Greece

- Director of 2nd Cardiology Department University Hospital of Ioannina

- Committee of education of the Greek NHS (substitute member)

- Vice President of the Medical School of Ioannina

7. Prof. Heinz Weber, MD, PhD:

Financial: 1

Nonfinancial: Member of the Executive of the UEMS Cardiology Section

Member of the EBAC Reviewing Committee

Chairman of the Council, ECSF

Scientific Committee, Austrian Academy for Physicians

8. Eugene Pozniak

Financial: 0

Nonfinancial: Programme Director, European CME Forum Managing Director, Siyemi Learning

Member, Editorial Board, JECME

Chair, Good CME Practice group

Board, Global Alliance for Medical Education

9. Robert Schäfer, MD

Financial: 0

Nonfinancial: CEO, EBAC

President, General Assembly German National Ass. for Occupational Health and Safety in Medicine and Welfare,

Member, Board of Children Safety Foundation NRW, Germany

Member, Board of Trustees KKLE Hospitals, Kleve, Germany

CEO Chamber of Physicians North-Rhine, Duesseldorf, Germany, retired

10. Graham T. McMahon, MD, MMSc

Financial: 0

Non-financial:

President and CEO, Accreditation Council for CME, Chicago, USA

11. Kate Regnier, MA, MBA

Financial: 0

Non-financial:

Executive Vice President, Accreditation Council for Continuing Medical Education (ACCME), Chicago

12. Helmut Koenig, Dipl.-Kfm., German Public Auditor, Tax Advisor

Financial: 0

Non-financial: Partner Beiten Burkhardt Lawyers, Duesseldorf, Germany

Chairman, VAT Working Group at Institute of Public Auditors in Germany

Member of Tax Compliance Working Group at Institute of Public Auditors in Germany

13: Samar Aboulsoud, MD, MBBCH, MSc Int Med, MSc Med Ed, FHEA, MAcadMED

Financial: 1

Non-financial:

Acting CEO, Qatar Council for Healthcare Practioners, Ministry of Public Helath, Doha, Qatar

14. Hans Gehle, MD

Financial: 0

Non-financial:

Bergmannsheil and Paediatric Hospital Buer, Gelsenkirchen

Chairman, Committee for Training and Education Politics, Marburger Bund, Berlin

15. Peter Mills MD, BM, BcH (oxon), BsC, MA, FRCP

Financial: 0

Non-financial:

Member, ECSF Board, Cologne/London

16. Prof. Daiana Stolz, MD, PhD

Financial: 3

Grants/research support: Thermo-Fisher Scientifics, Swiss National Foundation, Internal Medicine Research Grant, University Hospital Basel, ResMed, Weinmann AG, Pan Gas AG, Novartis, Boston Scientifics, Curetis

Speakers honoraria or consultations fees: Boehringer Ingelheim, Almirall, Novartis, Glaxo, Astra, Bayer

Non-financial:

Clinic for Pulmonary Medicine and Respiratory Cell Research, University Hospital Basel, Basel, Switzerland

Member of the steering board of the Swiss Respiratory Society, since 2010

Past president of the European Board of Accreditation in Pneumology (2014-2016)

Member of the Editorial Board CHEST, since 2010

Member of the Pneumology Board Committee, Swiss Respiratory Society, since 2013

Past Co-chair of the Training Centre Accreditation Committee, HERMES 2014-2016

Member of the Educational Committee, American Thoracic Society, 2015-2016

Member of Long Range Planning Committe, Assembly 10, European Respiratory Society, since 2015

Fellow of the College of Chest Physician, since 2015

Member of the European Respiratory Society Fellowships & Awards Working Group, since 2015

Chair, International Affairs, Swiss Respiratory Society, since 2015

Member of the Lung function Committee, Swiss Respiratory Society, since 2015

Chair, Organizing committe, CHEST/SGP 2017, Basel, Switzerland

President Post Graduate Training Committee, Swiss Pneumology Society, since 2016

Education Council elect, European Respiratory Society, since 2016

17. Prof. Gerd Antes, DSc

Financial:1

Non-financial:

Director, Cochrane Germany (formerly German Cochrane Center), University Medical Center,

Freiburg, Germany.

Member of the Guideline Committee of the AWMF (Arbeitsgemeinschaft der Wissenschaftlichen Medizinischen Fachgesellschaften)

Member of the German Section of the International Biometric Society

Member of the GMDS (Deutsche Gesellschaft für Medizinische Informatik, Biometrie und Epidemiologie e.V.)
